# Structural diversity and polymorphism in amyloid fibrils from single organs and single patients with AL amyloidosis

**DOI:** 10.1063/4.0001120

**Published:** 2025-10-27

**Authors:** Parker T Bassett, Lorena Saelices, Binh Nguyen, Virender Singh, Gareth Morgan

**Affiliations:** 1 UT Southwestern Medical Center, Dallas, TX, USA; 2 Boston University, Boston, MA, USA

## Abstract

Amyloidogenic light chain (AL) amyloidosis is a complicated disease that results from aggregation of antibody light chain proteins into amyloid fibrils, that then disrupt organ function. A major gap in AL studies concerns the structural landscape of AL amyloid fibrils and how fibril structures might relate to clinical phenotypes of those with the disease. Thus far, it has been shown that amyloid fibrils from a single patient have little to no structural variability, regardless of the organ amyloid is extracted from. Additionally, AL amyloid fibrils from different patients that have similar amino acid sequences have been shown to have related amyloid folds. We sought to expand on this previous work by using cryo-electron microscopy to determine the structures of ex vivo AL amyloid fibrils from multiple patients and multiple organs. We observed diverse amyloid structures from unique patients, regardless of sequence similarity to previous structures. Additionally, we observed evidence of multiple structural conformations and diverse structural ensembles within individual patients and organs. Taken together, our observations suggest that AL amyloid fibrils can adopt diverse structural folds in spite of sequence similarity, and that the local tissue environment can influence the deposition of a unique AL fibril structure. In sum, our work demonstrates alternative conclusions compared to previous studies, and points to a new, unexplored model for how AL amyloid structures may influence the disease phenotype.

